# Usefulness of circulating tumor DNA by targeting human papilloma virus-derived sequences as a biomarker in p16-positive oropharyngeal cancer

**DOI:** 10.1038/s41598-021-04307-3

**Published:** 2022-01-12

**Authors:** Ken Akashi, Toshihiko Sakai, Osamu Fukuoka, Yuki Saito, Masafumi Yoshida, Mizuo Ando, Takeshi Ito, Yoshinori Murakami, Tatsuya Yamasoba

**Affiliations:** 1 Department of Otolaryngology and Head and Neck Surgery, Graduate School of Medicine, University of Tokyo Hospital, 7-3-1 Hongo, Bunkyo-ku, Tokyo, 113-8655 Japan; 2 Department of Otolaryngology and Head and Neck Surgery, Kameda Medical Center, Chiba, Japan; 3 Department of Otolaryngology, Showa General Hospital, Tokyo, Japan; 4 Department of Otolaryngology-Head and Neck Surgery, Okayama University Graduate School of Medicine, Dentistry and Pharmaceutical Sciences, Okayama, Japan; 5 Division of Molecular Pathology, Institute of Medical Science, University of Tokyo, Tokyo, Japan

**Keywords:** Cancer, Biomarkers, Medical research, Oncology

## Abstract

In head and neck cancer, early detection of recurrence after treatment is important. The contemporary development of therapeutic agents have improved the prognosis after recurrence; however, no biomarker has been established for evaluating therapeutic effects or detecting recurrence. Recently, circulating tumor DNA (ctDNA), which comprises DNA derived from tumor cells and exists in the form of cell-free DNA in the blood, has attracted attention as a minimally invasive and repeatable biomarker for detecting cancer. We validated the usefulness of ctDNA of human papilloma virus (HPV)-derived sequences as a biomarker in HPV-related p16-positive oropharyngeal cancer by assessing 25 patients with p16-positive oropharyngeal cancer. Blood samples were collected from each patient at multiple time points during the treatment, and the plasma was preserved. The ctDNA was extracted from the plasma and analyzed using digital polymerase chain reaction. HPV-derived ctDNA was detected in 14 (56%) of the 25 patients. In all the patients, the samples were found to be ctDNA-negative after initial treatment. Cancer recurrence was observed in 2 of the 14 patients; HPV-derived ctDNA was detected at the time of recurrence. Our results indicate that HPV-derived ctDNA can be a prospective biomarker for predicting the recurrence of p16-positive oropharyngeal cancer.

## Introduction

Head and neck cancers are often diagnosed at the first visit as advanced cancers. Despite intensive treatment using multidisciplinary therapy, residual disease after the treatment and metastatic recurrence are common. Recently, such cases have been treated with molecular-targeted drugs and immune checkpoint inhibitors, which can prolong overall and progression-free survival and improve quality of life, making the early detection of recurrence more important^1^. However, there are no established biomarkers for determining the response to treatment or for detecting recurrence; therefore, the detection of recurrence still relies on follow-up by palpation and imaging^2^.

In recent years, circulating tumor DNA (ctDNA) has become a new biomarker for various cancers. ctDNA consists of DNA derived from tumor cells and exists in the blood in the form of cell-free DNA. The detection of ctDNA in blood not only enables quantitative estimation of the presence or absence of tumors and the degree of tumor progression but also provides genomic information such as specific genetic mutations in tumors. Because less than 10 mL of blood is sufficient for ctDNA analysis, it has attracted attention as a new biomarker that can be collected both noninvasively and repeatedly^3^.

Oropharyngeal cancer can be divided into two categories: one is a p16-negative tumor caused by loss of tumor suppressor gene, p16; the other is a p16-positive tumor caused by the integration of human papilloma viruses (HPVs), type 16 or 18. Given that HPV DNA is only integrated into p16-positive oropharyngeal cancer in the human body, exogenous HPV-derived DNA sequences can provide an ideal target for detecting ctDNA specific to the p16-positive oropharyngeal cancer.

In this study, we aimed to validate the usefulness of ctDNA by targeting HPV-derived DNA sequences as a biomarker in HPV-related, p16-positive oropharyngeal cancer. In p16-positive oropharyngeal cancer, HPV-derived ctDNA in patients with head and neck cancers has been reported using digital polymerase chain reaction (PCR)^4^, which is more sensitive than conventional real-time PCR.

## Methods

We included 25 patients diagnosed with p16-positive oropharyngeal cancer who underwent initial treatment in our department in the University of Tokyo Hospital between January 2017 and December 2018 and provided written informed consent. The patients comprised 22 men and 3 women, and the mean age was 66 years (range, 46–80 years; median, 66 years). The mean observation period was 15 months (range, 3–24 months; median, 16 months). As a negative control, we included four patients who had been diagnosed with nasopharyngeal cancer, which was not associated with HPV integration. This study was approved by the Ethics Committee of the University of Tokyo Hospital [Review No. G10112-(1)], and all the experiments were performed in accordance with relevant guidelines and regulations.

### Extraction of cell-free DNA from blood samples

At multiple time points during the course of treatment, we collected an additional 10 mL of blood in addition to samples for routine medical practice. Blood was collected in ethylenediaminetetraacetic acid (EDTA)-containing tubes and centrifuged at 3000 rpm for 10 min at 4 °C followed by 4000 rpm for 10 min within 4 h from blood collection. The supernatant of approximately 4 mL was stored as plasma at − 20 °C. We extracted 50 µL of cell-free DNA solution from 1 mL aliquots of plasma using the Maxwell^®^ RSC ccfDNA Plasma Kit (Promega).

### Digital PCR

We used the Taqman probe, which is typically employed in real-time PCR, and prepared the primers and probe sets targeting the specific regions of E6 and E7 of the HPV viral DNA which were integrated into cancer DNA (HPCL purification, Integrated DNA Technology, Inc.) (Table 1). We performed digital PCR using the QX200™ Droplet Digital™ PCR System (Bio-Rad). The reaction solution was prepared by mixing 8 µL aliquots of cell-free DNA solution extracted from plasma, 2 µL of primer and probe solution, and 10 µL of buffer and was converted into approximately 20,000 droplets floating in oil using a droplet generator (Bio-Rad). The PCR reaction was subsequently carried out in a T100™ Thermal Cycler (Bio-Rad); the fluorescence intensity of each droplet was measured and analyzed using a QX200™ Droplet Reader (Bio-Rad). If both E6- and E7-derived sequences were detected, it was judged as ctDNA positive. We used *the Ribonuclease P/MRP Subunit P30* (*RPP30*) gene as a control DNA sequence, and the ratio of the number of the droplets with E6-positive droplets divided by the number of *RPP30*-positive droplets was used as the ctDNA value.

**Table 1:** Primers and probes used for digital polymerase chain reaction.

HPV_E6	Primer	Fw: TCAGGACCCACAGGAGCG
		Rv: CCTCACGTCGCAGTAACTGTTG
	Probe	FAM-CAGAAAGTT-ZEN-ACCACAGTTATGCACAGAGCT-IABkFQ
HPV_E7	Primer	Fw: CCGGACAGAGCCCATTACAA
		Rv: CGAATGTCTACGTGTGTGCTTTG
	Probe	FAM-CGCACAACCGAAGCGTAGAGTCACACT-IABkFQ
*RPP30*	Primer	Fw: GATTTGGACCTGCGAGCG
		Rv: GCGGCTGTCTCCACAAGT
	Probe	HEX-TTCTGACCTGAAGGCTCTGCG-IABkFQ

*HPV* human papillomavirus.

We used the Fisher exact test and Kruskal–Wallis test to determine significant differences. A *p* value of < 0.05 was considered significant.

## Results

Among the 25 patients, 19 were at clinical stage I, 1 was stage II, 3 were stage III, and 2 were stage IV, according to the 8th edition of the American Joint Committee on Cancer/International Union against Cancer classification. Treatment primarily consisted of surgery for 11 patients and radiotherapy for 14 patients. Induction chemotherapy was performed in one of the surgical cases and in six of the radiotherapy cases. Complete response (CR) was achieved in all patients after the first treatment.

**Figure 1:**
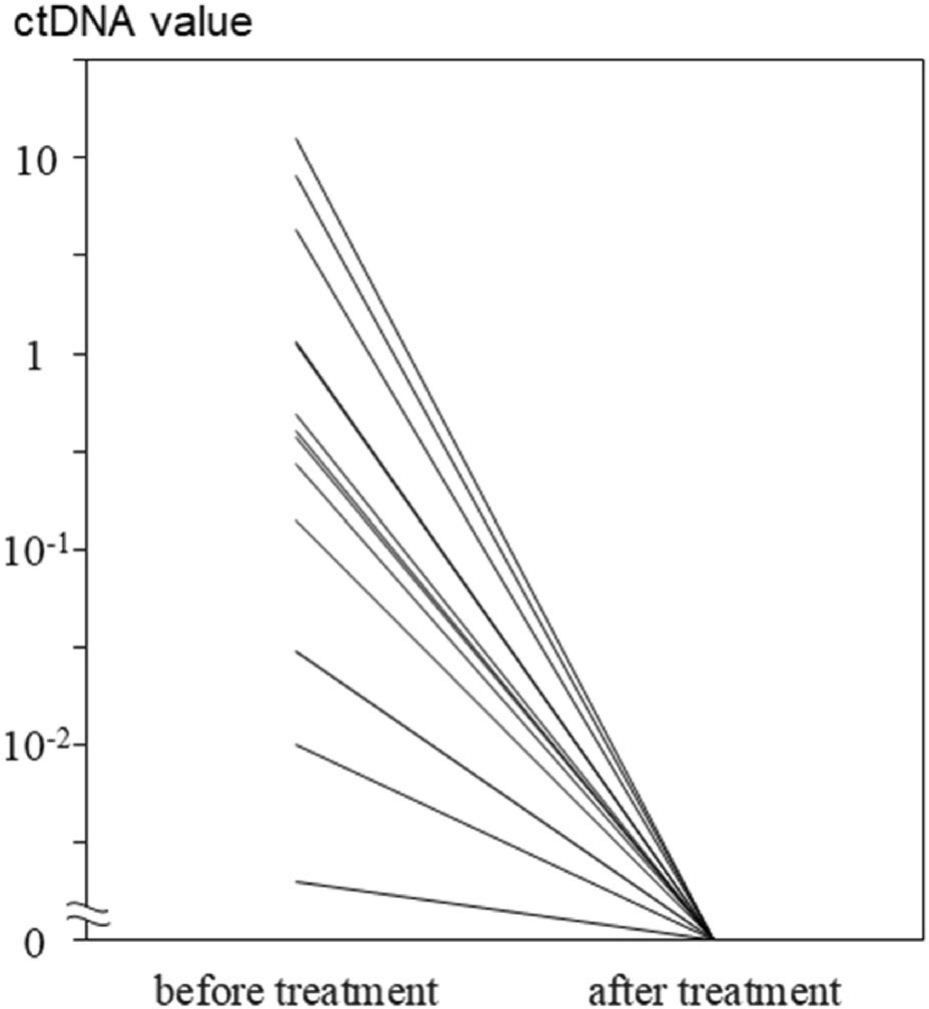
In all 14 pretreatment circulating tumor DNA (ctDNA)-positive cases, ctDNA became negative after initial treatment.

Pretreatment ctDNA was positive in 14 cases, with a positivity rate of 56%, and we found no significant differences in clinical indices such as clinical stage between ctDNA-positive and ctDNA-negative cases (Table 2). In all 14 pretreatment ctDNA-positive cases, ctDNA had become negative after treatment (Fig. 1). Among these cases, 2 developed a tumor recurrence, for whom ctDNA became positive again at the time of the recurrence. Positive and negative results in digital PCR for HPV-E6 and E7 amplification were consistent in all 25 cases. In all four negative control cases, ctDNA was negative.

**Table 2:** Clinico-pathological features of patients with p16-positive oropharyngeal cancer.

	Total	ctDNA		
		Positive	Negative	
	25	14	11	
**Sex**				
Male	22	13	9	n.s.*
Female	3	1	2	
**Age**				
≥ 65 years	17	9	8	n.s.*
< 65 years	8	5	3	
**Smoke**				
≥ 10 pack-years	15	8	7	n.s.*
< 10 pack-years	10	6	4	
**Alcohol**				
Habitual	14	8	6	n.s.*
Chance/none	11	6	5	
**T**				
T1	9	7	2	n.s.**
T2	11	4	7	
T4	5	3	2	
**N**				
N0	2	1	1	n.s.**
N1	19	9	10	
N2	4	4	0	
**Stage**				
I	19	10	9	n.s.**
II	1	1	0	
III	3	3	0	
IV	2	0	2	

*ctDNA* circulating tumor DNA, *n.s.* not significant.

*Fisher’s exact test; **Kruskal–Wallis test.

### Case presentation

#### Case 1: lateral wall cancer, T1N1, stage III

**Figure 2:**
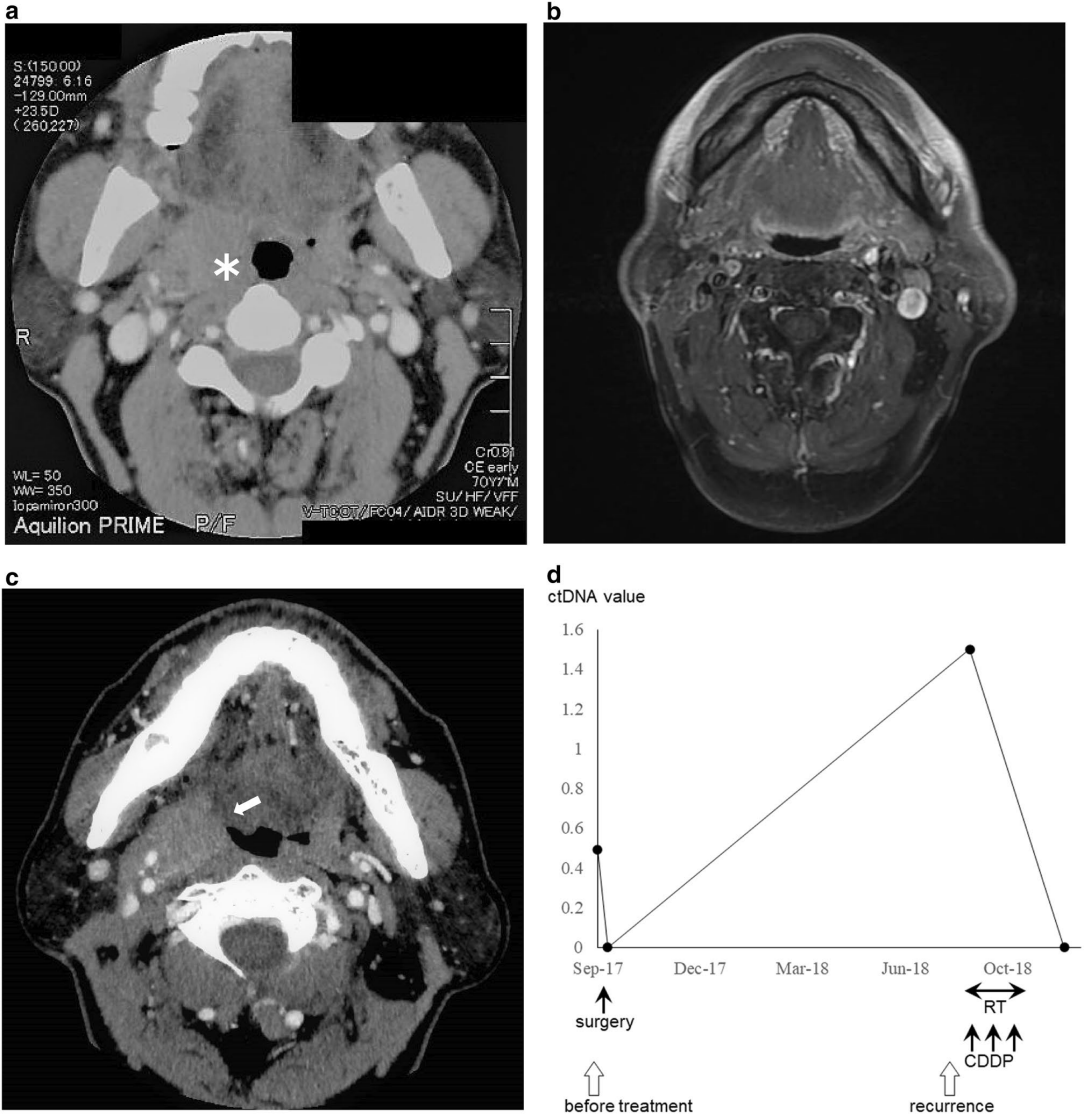
(**a**) Pretreatment computed tomography (CT) image shows the tumor (asterisk) at the right lateral wall of the oropharynx. (**b**) Magnetic resonance imaging scan after initial treatment shows complete response. (**c**) CT image shows the local recurrence of the tumor (arrow) at the right lateral wall of the oropharynx. (**d**) The pretreatment circulating tumor DNA (ctDNA) was positive and became negative after initial treatment with surgery. ctDNA was positive again at the time of local recurrence and turned negative again after concomitant chemoradiotherapy with CDDP.

This patient was ctDNA positive before the start of treatment and underwent resection of the primary lesion with neck dissection on the affected side in September 2017. Pathological examination indicated negative results for surgical margins and the absence of extranodal extension. The ctDNA became negative after this surgical treatment. The primary tumor recurred in August 2018, and ctDNA became positive again. Concomitant chemoradiotherapy with CDDP resulted in CR, and ctDNA became negative again (Fig. 2).

#### Case 2: lateral wall cancer, T4N2c, stage IV

**Figure 3:**
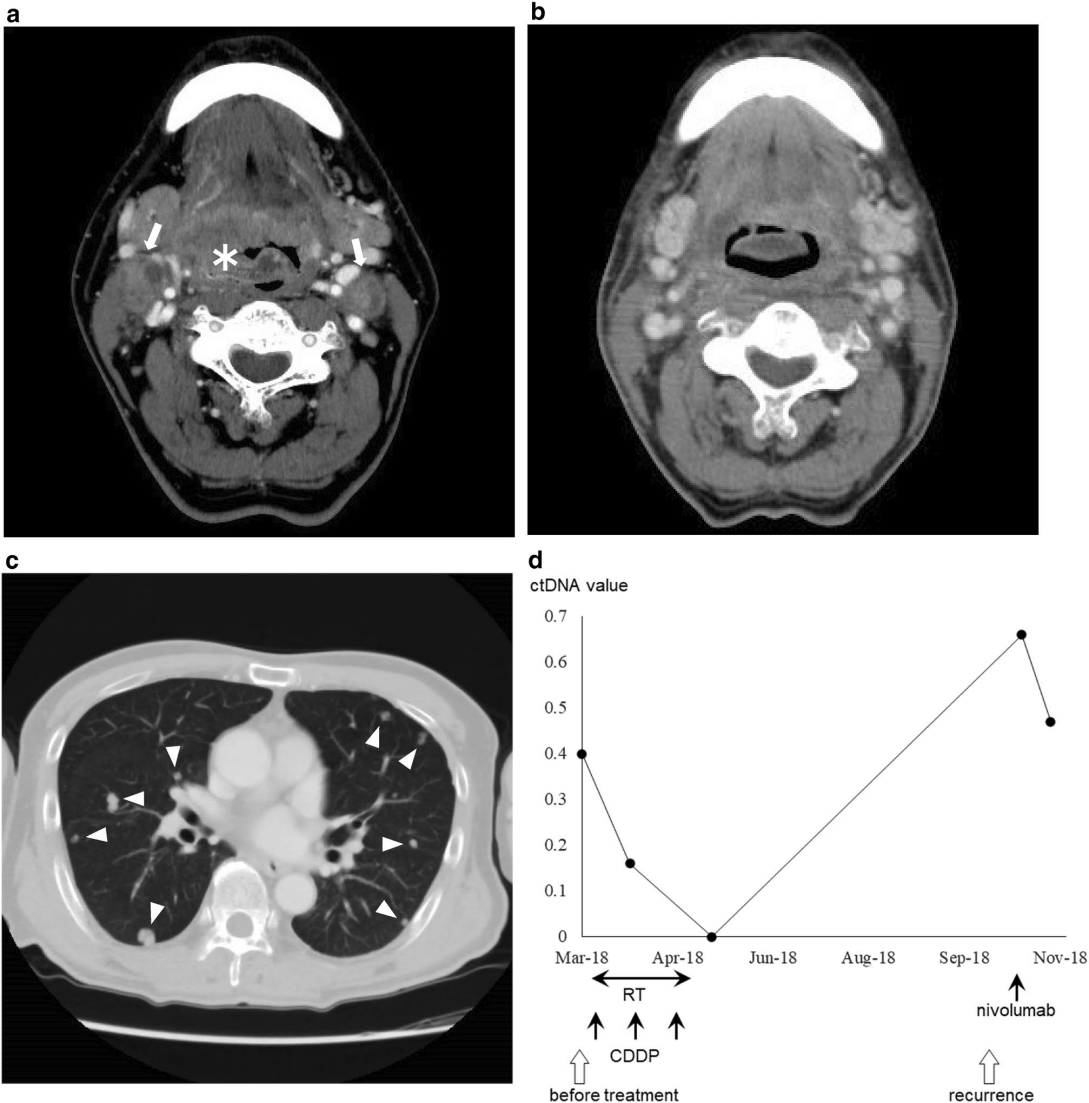
(**a**) Pretreatment computed tomography (CT) image shows the tumor (asterisk) extending from the right lateral wall to the anterior wall of the oropharynx and cervical node metastasis (arrows). (**b**) CT image after initial treatment shows complete response. (**c**) CT image shows multiple lung metastases (triangles). (**d**) The pretreatment circulating tumor DNA (ctDNA) was positive and turned negative after initial treatment with concomitant chemoradiotherapy with CDDP; ctDNA was positive again at the time of local and distant recurrence and remained positive despite the administration of immune checkpoint inhibitor therapy with nivolumab.

The patient was ctDNA positive before the start of treatment. In March 2018, the patient underwent concomitant chemoradiation with CDDP for a primary lesion that extended from the oropharynx to the larynx. At the end of treatment, ctDNA became negative. In October 2018, local recurrence was observed at the larynx and multiple lung metastases, and the ctDNA became positive again. Despite immune checkpoint inhibitor therapy with nivolumab, the disease was progressive, and the ctDNA remained positive (Fig. 3).

## Discussion

To investigate the usefulness of ctDNA as a biomarker in head and neck cancer, we measured HPV-derived ctDNA in p16-positive oropharyngeal cancer patients during the course of treatment and compared the results to the clinical course. ctDNA was detected in 14 of 25 (56%) patients, and all patients became negative for ctDNA after the initial treatment. Relapse occurred in 2 of 14 patients, and ctDNA was detected in the blood at the time of relapse for both patients, indicating that ctDNA changes reflected the clinical course of these patients.

Biomarkers are biological substances, such as proteins and DNA, found in tissues or body fluids (e.g., blood and urine) that are correlated with the state of disease and serve as indicators of treatment response. For malignant tumors, biomarkers are used clinically in one of two ways: (1) for the prediction of the treatment effect and (2) for the evaluation of disease activity. As an example of the former, the presence or absence of an epidermal growth factor receptor (EGFR) mutation is used to predict the efficacy of EGFR antagonists in non-small-cell lung cancer^5^; for the latter, prostate-specific antigen levels in the blood are used to evaluate disease activity in prostate cancer^6^. In head and neck cancers, the neutrophil-to-lymphocyte ratio^7^, albumin level, and C-reactive protein level in the blood have recently attracted attention as biomarkers for the prediction of therapeutic efficacy and prognosis but are currently used only for reference. The blood thyroglobulin level in papillary thyroid carcinoma and blood calcitonin and carcinoembryonic antigen level in medullary carcinoma are useful biomarkers for the evaluation of disease activity, but no effective biomarker has been established for squamous cell carcinoma.

The importance of ctDNA has attracted attention since 1994, when DNA fragments with *RAS* mutations were detected in the blood of patients with various cancers^8,9^. The *KRAS* mutations in pancreatic cancer and the *KRAS* and *TP53* mutations in colorectal cancer have been detected in ctDNA, and clinical applications are presently underway^10^. Sequencing analysis can also be performed, which not only predicts the tumor’s quantitative state but also provides genomic information. When multiple cancer lesions are present, each lesion may contain a differently altered tumor genome, and biopsies performed from a single location may provide biased information. In addition, the tumor genome is known to change during the course of treatment, but repeated tissue biopsies are invasive and are associated with a high burden. ctDNA provides comprehensive genomic information of the entire tumor and is easy to repeat; thus, it has attracted considerable attention as a liquid biopsy.

Weng et al*.* reported that HPV-derived ctDNA was detected in 41 of 47 (87%) cases of head and neck squamous cell carcinoma and in 18 of 21 (86%) cases of HPV-related cancer^11^. Chera et al*.* reported that HPV-derived ctDNA was detected in 84 of 103 (82%) HPV-related oropharyngeal cancers^12^. In the current study, the sensitivity of ctDNA was 56%, which was lower than those reported by the two aforementioned studies. However, after the completion of the first treatment, all the positive cases became negative, and repositivity was detected at the time of recurrence, suggesting that ctDNA is useful as a biomarker. The storage conditions of the blood samples might be a potential cause for the low sensitivity. In the present study, blood collection for ctDNA and regular clinical examination was often performed early in the morning, and in some cases, a maximum of 4 h had passed before the collected blood was stored as plasma, although the half-life of ctDNA is shown to be relatively short, ranging from 16 min to 2.5 h^13^. Furthermore, as a result of destruction, the intracellular DNA of normal blood cells might have leaked into the specimen, making trace amounts of ctDNA undetectable. Because the sensitivity of ctDNA was lower than expected, we attempted to shorten the time from blood collection to processing as much as possible in the latter half of the study period. In the first half of the study period, 6 of 13 (47%) specimens were positive for ctDNA, whereas in the second half, 8 of 12 (67%) specimens were positive, which suggests the importance of the time required for specimen processing. Based on previous reports, we used blood collection tubes containing EDTA, which are used clinically for blood typing; however, we expect the detection rate to be improved with the use of tubes specified for cell-free DNA extraction. These tubes might not affect specimens that are processed immediately after collection in research phases substantially, but for clinical applications, the use of dedicated blood collection tubes is desirable because of the time elapsed from blood collection to storage.

ctDNA analysis can be broadly divided into two methods: one by PCR and the other by sequencing analysis. The digital PCR used herein is an analytical method that applies the PCR reaction, and it has attracted attention as a third-generation PCR that enables absolute quantification following conventional PCR for qualitative analysis and real-time PCR for relative quantification^14^. The PCR reaction is performed after dividing the reaction solution in which the primer and probe are mixed into many micro compartments of approximately 20,000 by transforming the solution into fine droplets. By keeping the concentration of the template DNA sufficiently low, each compartment contains one or zero molecules of DNA, and the amplification reaction occurs only within the droplets that contain the target sequence, and fluorescence is emitted. By analyzing the fluorescence intensity of all the droplets and measuring the percentage of positive droplets, the concentration of the target can be quantified in absolute terms. In particular, with respect to mutations of a low frequency, it is difficult to detect mutations of less than 1% using conventional real-time PCR; however, with digital PCR, it is possible to detect mutations of 0.001%, which is useful for ctDNA analysis that exists in only small amounts^15^.

The use of sequencing analysis can provide not only quantitative information but also genomic information, as described previously^16,17^. PCR-based methods require knowledge regarding the tumor-specific genetic abnormalities to be targeted. In cases of head and neck cancers in which there are almost no specific genetic mutations except for p16-positive oropharyngeal and Epstein–Barr virus (EBV)-positive nasopharyngeal cancers, identifying genetic abnormalities is necessary. However, this is not required in sequencing analysis, and the creation of primers and probes is also not needed, which makes the testing process considerably simple. Nevertheless, the cost of sequencing analysis is high, obtaining results requires a substantial amount of time, and specialized knowledge is required to analyze the obtained data.

In this study, we aimed to establish a new biomarker for head and neck cancer using digital PCR. We targeted p16-positive oropharyngeal cancer, for which the target gene abnormality has been established and detected in blood. EBV-positive nasopharyngeal cancer and thyroid cancer would be similarly applicable. We expect ctDNA to be applied in the future to other head and neck cancers through the use of sequencing analysis to identify and target individual tumor-specific genetic abnormalities.

## Data Availability

The datasets generated during and/or analyzed during the current study are available from the corresponding author upon reasonable request.
